# Monitoring for compliance with a ketogenic diet: what is the best time of day to test for urinary ketosis?

**DOI:** 10.1186/s12986-016-0136-4

**Published:** 2016-11-04

**Authors:** Paul Urbain, Hartmut Bertz

**Affiliations:** Department of Medicine I, Section of Clinical Nutrition and Dietetics, Medical Center – University of Freiburg, Hugstetterstr 55, 79106 Freiburg, Germany

**Keywords:** Ketogenic diet, Ketosis, Ketone bodies, Ketonuria, Urine testing, Diet compliance

## Abstract

**Background:**

The ketogenic diet (KD) is a very low-carbohydrate, high-fat and adequate-protein diet with no calorie limit that induces a metabolic condition called “physiological ketosis”. It was first introduced to treat epilepsy in the 1920s and has become quite popular recently as weight-loss and performance-enhancing diet. Its therapeutic use in a range of diseases is under investigation. During KD interventions people are supposed to monitor compliance with the dietary regimen by daily urine testing for ketosis. However, there are no studies investigating the best time for testing.

**Findings:**

Twelve healthy subjects (37 ± 11 years; BMI = 23.0 ± 2.5 kg/m^2^) were instructed to, during the sixth week of a KD and with stable ketosis, measure their urine (8×) and blood (18×) ketone concentration at regular intervals during a 24-h period. According to their 1-day food record, the subjects consumed on average a diet with 74.3 ± 4.0 %, 19.5 ± 3.5 %, and 6.2 ± 2.0 % of total energy intake from fat, protein and carbohydrate, respectively. The lowest blood ß-hydroxybutyrate (BHB) (0.33 ± 0.17 mmol/l) and urine acetoacetate (AA) (0.46 ± 0.54 mmol/l) concentrations were measured at 10:00, respectively. The highest BHB (0.70 ± 0.62 mmol/l) and AA concentrations were noted at 03:00, respectively. Via urine testing the highest levels of ketosis were found at 22:00 and 03:00 and the highest detection rates (>90 %) for ketosis were at 07:00, 22:00 and 03:00, respectively.

**Conclusions:**

These results indicate that ketonuria in subjects with stable ketosis is highest and can be most reliably detected in the early morning and post-dinner urine. Recommendations can be given regarding precise time of the day for measuring ketone bodies in urine in future studies with KDs.

## Introduction

The ketogenic diet (KD) is a very low-carbohydrate (<30–40 g/day, ≈ 5 % of energy), high-fat (>60 % of energy) and adequate-protein diet that without limiting calories (eucaloric) induces a metabolic condition called “physiological ketosis”, thus distinguishing it from the severe pathological ketosis (ketoacidosis) often observed in uncontrolled diabetes [1, 2]. The lower insulin level via low carbohydrate intake raises the serum glucagon level and induces lipolysis, leading to the augmented availability of fatty acids [3]. These are metabolised by the mitochondria of liver cells to two water-soluble types of ketones (ketogenesis): acetoacetate (AA) and ß-hydroxybutyrate (BHB) [4]. A third ketone body, acetone, is produced by the enzymatic decarboxylation of AA, and is largely exhaled unused. AA and BHB become the primary energy source for the brain and other tissues [4].

For the optimal clinical management of children with epilepsy on a KD, the International Ketogenic Diet Study Group recommends monitoring compliance by urine testing for ketosis several times a week [5]. Except for one study that did not test for ketosis [6], the majority took daily measurements of urinary ketone bodies without [7–9] or with contrary recommendations on the precise time of the day for measuring: 8:00 [10] or in the afternoon [11]. Furthermore, a study in healthy adults who consumed four ketogenic meals over 12 h revealed that intra-day blood and urinary concentrations of ketone bodies fluctuate strongly [12] and can be affected by several factors [13].

At the beginning of our KetoPerformance study in healthy adults investigating the impact of a 6-week KD without caloric restriction on physical performance, we were unable to give concrete advice to our subjects concerning when it is best to test for the presence of ketosis in urine during the day. Therefore, we aimed to investigate in a portion of the study population the intra-day course of blood and urinary concentrations of ketone bodies during a KD.

## Methods

### Subjects

The KetoPerformance study with its before-and-after comparison design was registered at germanctr.de as DRKS00009605 and took place from February to June 2016. Exclusion criteria included underweight, obesity, kidney stones, pregnancy, diabetes mellitus and any fatty acid-metabolism disorders. The study protocol was approved by the Ethics Commission of the Albert-Ludwig University Freiburg (494/14) and all subjects signed a written consent form. Twelve of the 42 subjects from the KetoPerformance study could be recruited for the present substudy.

### Experimental design and dietary intervention

The experimental intervention consisted of a KD without caloric restriction lasting 6 weeks with a previous preparation period including detailed instructions during teaching classes and individual counselling by a dietitian. The subjects were free to follow a KD according to their personal preferences but were advised to reach a ratio by weight of approximately 1.8:1 fat to carbohydrate and protein combined, yielding a diet with 80, 15, and 5 % of total energy intake from fat, protein and carbohydrate, respectively.

During the KD intervention's sixth week, our substudy subjects were instructed to measure urine and blood ketone concentrations at regular intervals in as close proximity as possible during a 24-h period from 07:00 to 07:00 in the morning. During the day (07:00 till 22:00) blood and urinary ketones were measured every full hour and every three hours, respectively. During the night, blood and urinary ketones were measured once at 03:00. In total blood and urine and ketones were measured 18 and 8 times, respectively, and were recorded in a table sheet. Subjects were asked to drink 400 ml of water every 3 h during the day to ensure sufficient urination and to keep detailed food diaries and exercise logs throughout the 24-h study period.

### Measurements of ketone concentrations

Urine ketone concentrations were measured using over-the-counter reagent strips (Ketostix, Bayer Vital GmbH, Leverkusen, Germany), which determine the presence of AA upon reaction with nitroprusside salt. The end of the strip was passed through the urine stream and the colour then compared to the colour chart provided with the product 15 s after the reaction.

Blood ketone concentrations were measured using an electrochemical capillary blood monitor device (FreeStyle Precision Neo H, Abbott, Wiesbaden, Germany) with the corresponding individually foil-wrapped test strips for BHB. Each test strip contains three electrodes (working, reference, and fill trigger) and the test is automatically initiated upon the application of enough blood. Subjects were advised to clean and dry their fingertips before each prick with the single-use lancing devices provided with three depth settings (Accu-Chek, Roche Diagnostics GmbH, Mannheim, Germany). After insertion of a test strip into the device, a drop of blood was applied to the assigned spot and the BHB concentrations were read from the display and recorded.

### Energy and nutrient intake

Two semi-quantitative 7-day food records were obtained from all subjects before and during the last week of the 6-week KD intervention. Precise oral and written instructions were given individually from the dietitian on how to accurately record the amounts and types of food and beverages. Subjects were given a digital portable scale (KS 22, Beurer GmbH, Ulm, Germany) and instructed to weigh all food items separately if possible or to estimate the amounts and take a photograph. The energy and macronutrient intake of the 1-day food records were analysed by a dietitian with a nutritional database software (Prodi 6.5 basis, Nutri-Science GmbH, Stuttgart, Germany).

### Physical activity

Subjects recorded all their physical activities (leisure time and sports) other than basic activities. Total additional physical activities were expressed by their rate of energy expenditure in metabolic equivalent of task (MET) based the reference data published by Ainsworth et al. [14]. One MET for a reference adult is approximately 1 kcal*kg/h.

### Statistics

We used IBM SPSS 22 for statistical analysis (IBM, NY, USA). All variables were normally distributed (Kolmogorow-Smirnow test) and are presented as means ± standard deviations. Paired *t*-tests were used to compare means. Results with *P* <0.05 will be denoted as significant.

## Results

### Characteristics of the subjects

All subjects recruited completed the substudy. Characteristics of the study population are summarised in Table 1. Mean age and body mass index of the subjects were 37.5 ± 11.1 years and 23.0 ± 2.5 kg/m^2^, respectively. Seven subjects reported energy expenditure during the substudy from additional physical activities ranging from 2.3 to 15.7 MET out of one to five activities.

**Table 1:** Characteristics of the subjects and energy expenditure from additional physical activities (*n* = 12)

Subject	Sex	Age (years)	Weight^a^ (kg)	BMI (kg/m^2^)	MET (amount of physical activities)
1	M	35	89.7	25.6	0
2	F	27	58.2	20.1	0
3	M	44	74.9	22.9	15.7 (2)
4	F	35	54.8	19.6	0
5	F	47	74.1	25.9	0
6	M	57	65.7	23.8	0
7	M	24	82.8	26.7	9.4 (3)
8	F	33	61.8	21.9	2.3 (2)
9	F	28	59.3	20.3	6.3 (5)
10	F	42	77.8	25.7	10.0 (2)
11	F	53	74.4	23.0	7.0 (1)
12	F	25	62.2	20.8	3.5 (1)
Mean ± SD	4 (33.3) : 8 (66.7)^b^	37.5 ± 11.1	69.6 ± 10.9	23.0 ± 2.5	4.5 ± 5.2

*Abbreviations*: *BMI* body mass index, *F* female, *M* male, *MET* metabolic equivalent of task

^a^Measured at inclusion to main KetoPerformance study

^b^Male : female n (%)

### Diet compositions

Macronutrient compositions of the habitual diet (before KD), intervention diet and diet of the substudy day are shown in Table 2. The average intake of all three macronutrients during the KD period was significantly different from the habitual diet. The substudy day was representative of the KD period, as macronutrient composition did not differ from the 7-day food record at the end of the KD. According to their 1-day food record, the subjects consumed on average a diet with 74.3 ± 4.0 %, 19.5 ± 3.5 %, and 6.2 ± 2.0 % of total energy intake from fat, protein and carbohydrate, respectively.

**Table 2:** Macronutrient intake assessed using semi-quantitative food records

Subject	Carbohydrates (% of total energy intake)			Protein (% of total energy intake)			Fat (% of total energy intake)		
	Before KD^a^	At the end of KD^a^	Substudy day^b^	Before KD^a^	At the end of KD^a^	Substudy day^b^	Before KD^a^	At the end of KD^a^	Substudy day^b^
1	42.8	8.5	6.4	13.2	20.9	19.7	44.1	70.6	74.0
2	47.3	6.6	6.0	12.9	25.3	19.5	39.8	68.1	74.5
3	47.9	5.7	8.0	12.8	20.3	19.8	39.3	74.0	72.2
4	47.5	5.8	6.3	13.7	14.2	17.4	38.8	80.1	76.3
5	51.3	12.8	9.1	12.3	20.9	23.8	36.4	66.3	67.1
6	42.8	7.9	3.6	16.5	17.9	21.1	40.7	74.1	75.3
7	50.4	6.3	5.4	15.4	24.6	26.8	34.2	69.1	67.8
8	54.1	4.4	5.1	14.0	16.5	12.8	31.9	79.1	82.1
9	49.7	8.1	6.0	14.9	19.8	19.3	35.4	72.2	74.8
10	44.6	6.7	3.3	15.0	17.7	19.6	40.4	75.6	77.1
11	37.8	8.3	5.2	14.9	19.6	17.8	47.2	72.0	77.0
12	46.4	10.7	9.7	15.6	17.7	16.3	38.0	71.6	73.9
Mean ± SD	46.9 ± 4.4	7.6 ± 2.3	6.2 ± 2.0	14.3 ± 1.3	19.6 ± 3.2	19.5 ± 3.5	38.8 ± 4.2	72.7 ± 4.1	74.3 ± 4.0
*P* ^*c*^	<0.001			<0.001			<0.001		
		0.027			0.883			0.079	

^a^mean of 7-day food record

^*b*^mean of 1-day food record

^*c*^ *t*-test for paired variables

### Course of ketone bodies in blood and urine

**Fig. 1:**
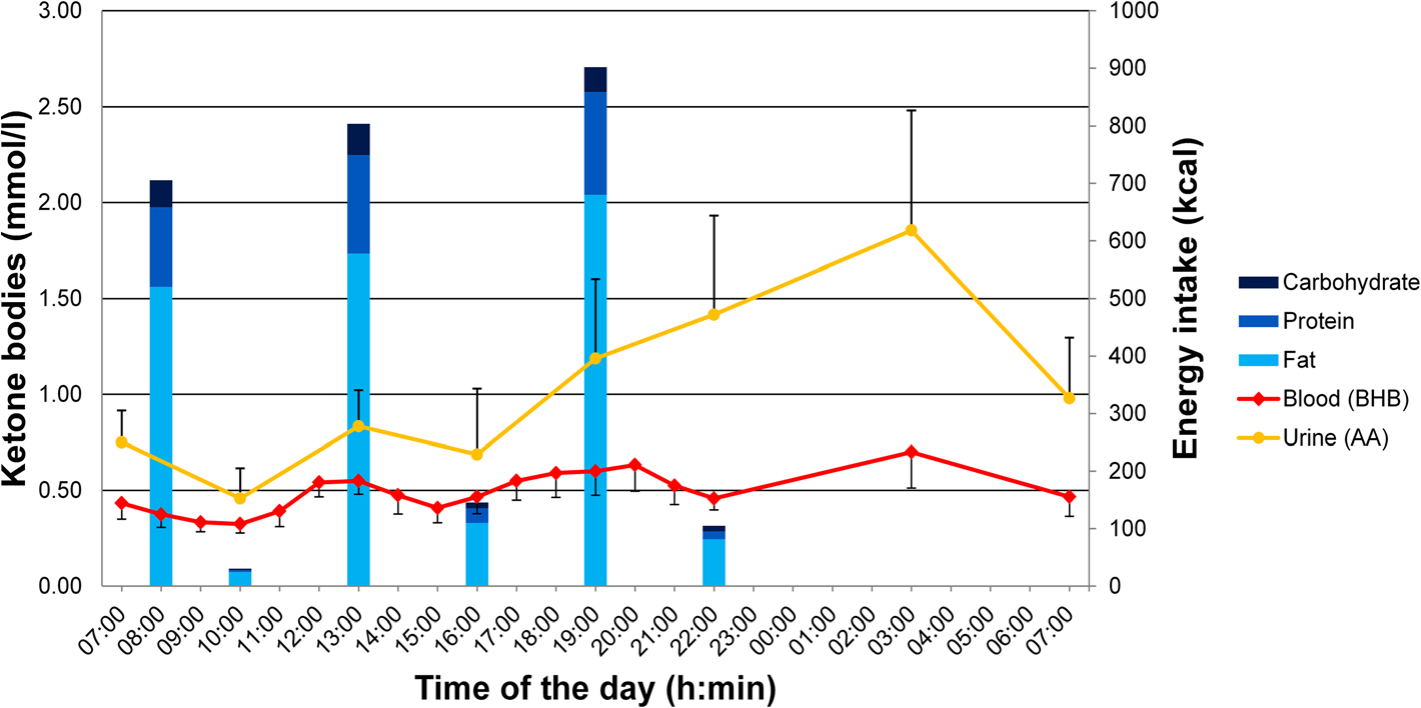
Course of ketone bodies in blood and urine (mmol/l) during the 24-h period and energy intake. *Blue* bars represent the energy (kcal) and macronutrient compositions (% of energy intake) of the principal meals and snacks. Error bars represent 2 standard errors. Mean times for the principal meals were as follow: breakfast 07:40 ± 0:30; lunch 12:53 ± 0:30; dinner 19:08 ± 0:40. Mean deviations in time to full hour of blood and urine measurements were 3.6 ± 7.9 min and 2.9 ± 7.8 min, respectively

Besides the course of ketone body concentrations measured in blood and urine, the mean macronutrient composition (% of total energy intake) of the principal meals and snacks are displayed in Fig. 1. At the beginning, fasting blood BHB and urine AA concentrations measured at 07:00 were 0.43 ± 0.29 mmol/l and 0.75 ± 0.57 mmol/l. The lowest (0.33 ± 0.17 mmol/l) and highest (0.70 ± 0.62 mmol/l) blood BHB concentrations were measured at 10:00 and 03:00, respectively. The lowest (0.46 ± 0.54 mmol/l) and highest (1.85 ± 2.17 mmol/l) urine AA concentrations were noted at 10:00 and 03:00, respectively, also.

**Fig. 2:**
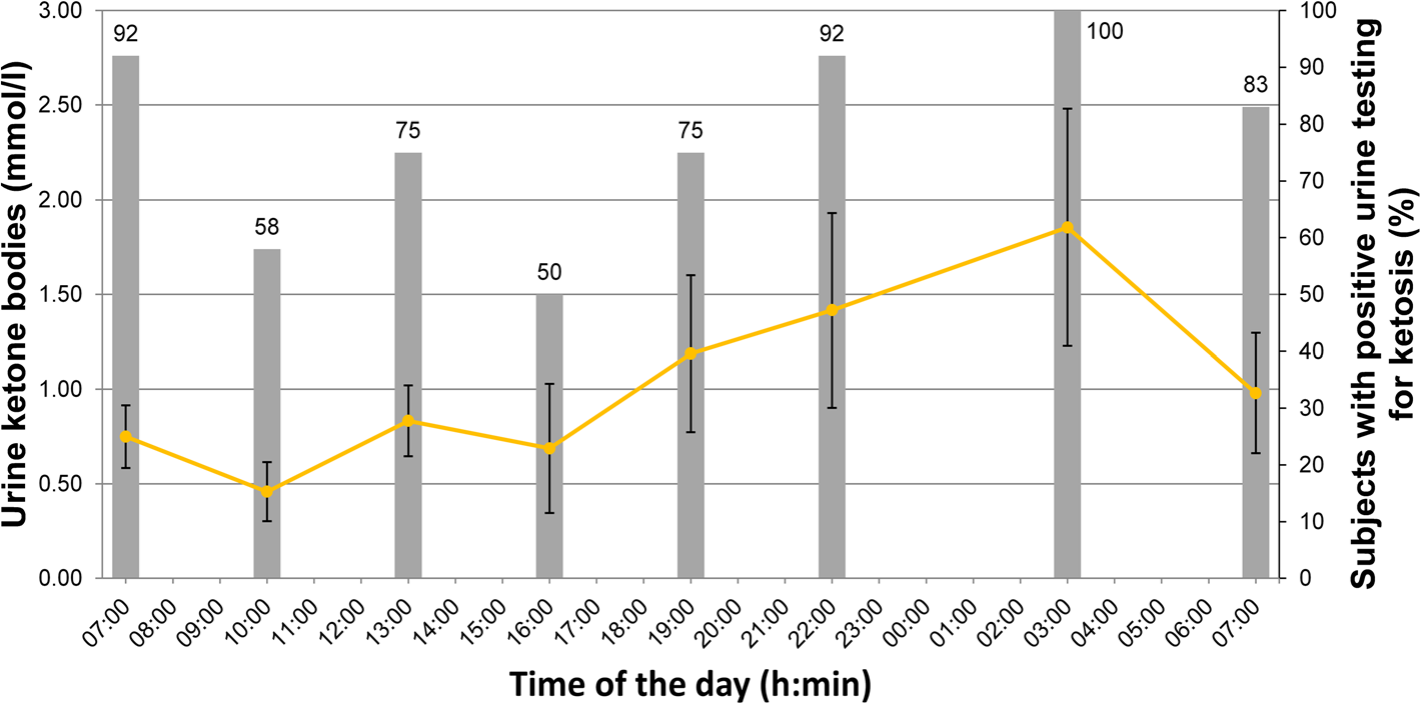
Course of urine ketone bodies (mmol/l) during the 24-h period and % of subjects with positive urine testing for ketosis via reagent strips (*grey bars*). Error bars represent 2 standard errors. Mean deviation in time to full hour of urine measurements was 2.9 ± 7.8 min

Figure 2 illustrates the course of urinary ketone body concentrations with positive urine testing for ketosis via urine reagent strips. The highest detection rates (>90 %) for ketosis were at 07:00, 22:00 and 03:00, respectively. The lowest detection rates (in just 58 and 50 % of the subjects) were measured at 10:00 and 16:00, respectively.

## Discussion

Regular urine testing for ketosis is the most common and feasible approach to monitoring a KD's adherence, and it should be an essential factor in all low-carbohydrate and KD intervention studies. Daily self-testing of urinary ketones is the norm in the literature [7, 9–11, 15–17], except for one study investigating the capacity for moderate endurance exercise after a KD, where urine ketones were measured twice daily [18]. A few others did collect 24-h urine [19, 20], while others only measured blood ketones [21, 22] or did not even test for ketosis [6]. The International KD Study Group on the optimal clinical management of children following a KD recommends urine ketosis evaluation routinely, several times per week, but they give no advice on when to measure [5]. Hence, studies in children with medically refractory epilepsy treated with a KD had the families check urine ketones daily and thus also without specifying the time of day [7, 9, 16]. KD studies in adults without epilepsy usually did not address this issue either [8, 17, 18]. There is widespread disagreement regarding measurements of blood BHB or urinary AA in epilepsy patients, when beside diet compliance its efficacy should be monitored. Two studies found that blood BHB correlated better with seizure control than urinary testing of AA [7, 23]. However, the present trial was not designed to investigate this issue. An important finding of our study is that the course of blood and urine ketone bodies was very similar throughout the period of 24 h.

We identified just two publications giving recommendations on the precise time of the day for subjects to measure [10, 11]. The present substudy reveals that ketonuria in subjects during the sixth week of a KD and with stable ketosis can be most reliably detected in the first morning urine and several hours after dinner late in the evening. The slightly inferior detection rate of the second morning urine could be because subjects were obliged to urinate already 4 h previously at 3:00 during the night, yielding a 100 % detection rate. These results endorse the 8:00 recommendation to healthy adults put on a 6-week KD made by Sharman et al. [10].

The least favourable period of time according to our data was from 10:00 till 19:00, which stands in contrast to the recommendation by Klement et al. [11] (namely to use the urine reagent strips preferably in the afternoon). The considerably higher false negative rate during daytime measurements could be due to two opposing reasons: (1) meals and (2) physical activity. We detected continuous decreases in blood BHB and urinary AA lasting up to 3 h after each principal meal, except for urinary AA increasing after dinner. This concurs with the results in healthy adults published by Musa-Veloso et al. [12], where the time elapsed after each ketogenic meal revealed a significant negative effect on blood BHB and AA concentrations and that blood ketone bodies are rapidly metabolised for energy. Schwartz et al. [24] obtained similar results and advise children with epilepsy to consume more frequent meals and snacks to keep blood ketone concentrations more stable and to avoid the acute rises and falls resulting from ketogenic meals. Some (≈40 %) of our subjects performed no additional physical activities, whereas the others engaged in activities of light to vigorous intensity, factors that may also influence temporary daytime fluctuations in blood and urinary ketones, as muscles consume this energy source rapidly [25].

One of our study’s limitations is that we did not measure initial concentrations of ketone bodies under the usual high carbohydrate diet conditions. A further limitation is our study population's wide variation in additional physical activities.

However, our study has several strengths, including the eucaloric character of the diet to eliminate the confounding influence of negative energy balance and the synchronisation of the times of equal fluid intake volumes and food intake. A further strength is that the substudy was conducted during a KD's the sixth week, thus assuring a metabolic shift to stable ketosis and a study period outside the metabolic adjustment period lasting several days upon the initiation of such a diet.

In summary, our results suggest that urine testing for ketosis in the scope of monitoring compliance with a KD in adults is best done in the early morning urine and several hours after dinner late in the evening. Hence study participants in future studies with KDs can be given recommendations on the precise time of the day for measuring ketone bodies in urine. Our results should only apply with caution to children with epilepsy, as additional research is needed for this patient cohort.

## Abbreviations

AA: Acetoacetate
BHB: ß-hydroxybutyrate
BMI: Body mass index
KD: Ketogenic diet
MET: Metabolic equivalent of task
